# Refining Knowledge of Factors Affecting Vitamin B_12_ Concentration in Bovine Milk

**DOI:** 10.3390/ani11020532

**Published:** 2021-02-18

**Authors:** Mélissa Duplessis, Annie Fréchette, William Poisson, Lya Blais, Jennifer Ronholm

**Affiliations:** 1 Agriculture et Agroalimentaire Canada, Centre de Recherche et Développement de Sherbrooke, 2000 rue College, Sherbrooke, QC J1M 0C8, Canada; 2 Regroupement FRQ-NT Op+Lait, 3200 rue Sicotte, Saint-Hyacinthe, QC J2S 2M2, Canada; annie.frechette.2@umontreal.ca (A.F.); jennifer.ronholm@mcgill.ca (J.R.); 3 Département de Pathologie et Microbiologie, Faculté de Médecine Vétérinaire, Université de Montréal, 3200 rue Sicotte, Saint-Hyacinthe, QC J2S 2M2, Canada; 4 Département des Sciences Animales, Université Laval, 2425 rue de l’Agriculture, Québec, QC G1V 0A6, Canada; william.poisson.1@ulaval.ca; 5 Département de Microbiologie et D’infectiologie, Université de Sherbrooke, 2500 Boul. De l’Université, Sherbrooke, QC J1K 2R1, Canada; lya.blais@usherbrooke.ca; 6 Department of Food Science and Agricultural Chemistry, Faculty of Agricultural and Environmental Sciences, Macdonald Campus, McGill University, 21111 Lakeshore, Sainte-Anne-de-Bellevue, QC H9X 3V9, Canada; 7 Department of Animal Science, Faculty of Agricultural and Environmental Sciences, Macdonald Campus, McGill University, 21111 Lakeshore, Sainte-Anne-de-Bellevue, QC H9X 3V9, Canada

**Keywords:** cyanocobalamin, environmental factor, cattle, milk quality, bovine milk

## Abstract

**Simple Summary:**

Milk is considered a staple and complete food that contains several essential nutrients for humans. For instance, it is an excellent natural source of vitamin B_12_ (B12) due to the presence in the bovine rumen of a myriad of bacteria and archaea capable of producing the vitamin. This vitamin is only produced by prokaryotic microorganisms; vegetal products do not naturally contain it. A 250-mL glass of milk contains about 46% of the daily recommended dietary allowance of B12 for individuals over 13 years old. However, B12 concentration is variable in milk; therefore, identifying factors contributing to its variation is critical to ensure a stable B12 supply for consumers. The aims of these experiments are to gather more knowledge on possible sources of variation in B12 concentrations in milk in order to optimize and stabilize its levels and thereby improve the perception of milk in terms of its health benefits. We observed that B12 concentration increases when the conditions of the rumen are optimal, such as with elevated pH. We also studied if bedding type—e.g., recycled manure solid bedding or straw, which has been reported to impact milk microbiota—could have an impact on milk B12 concentration. In this study, no such correlation was detected. This paper is one of a series seeking to elucidate factors responsible for variations in milk B12 concentration.

**Abstract:**

Milk is an excellent source of vitamin B_12_ (B12) for humans. Therefore, being able to guarantee a high and consistent concentration of this vitamin would enhance consumer perception of milk as a health food. The aim of the paper was to gather additional knowledge on factors that could explain B12 variation in cow milk through two observational studies: (1) to explore the relationship between milk B12 and ruminal conditions, such as pH and volatile fatty acid concentrations; and (2) to examine the impact of bedding on B12 concentrations in bulk tank milk. For study 1, a total of 72 milk and ruminal liquid samples were obtained from 45 Holstein cows fitted with ruminal cannula between 10 and 392 days of lactation. For study 2, bulk tank milk samples were obtained from 83 commercial herds; 26 herds used recycled manure solid bedding and 57 used straw bedding. Milk samples were analyzed for B12 using radioassay. Using principal component regression analysis, we observed that ruminal pH and the acetate:propionate ratio for cows receiving the early lactation ration were positively correlated with milk B12. Bedding did not influence milk B12 in bulk tanks, which averaged 4276 pg/mL. In conclusion, as B12 is synthesized by ruminal bacteria, optimizing ruminal conditions had a positive effect on milk B12, while bedding management had no influence.

## 1. Introduction

As early as the 1980s, Canadian and American reports indicated that overall fluid milk consumption per capita was decreasing [1,2]. The same declining trend is continuing in many countries including Canada, USA, and Switzerland [3]. One explanation is the increasing consumption of nondairy, plant-based beverages which are available on the market. A survey conducted on consumers of plant-based beverages which sought to understand the decreasing consumption of dairy products indicated that drinkers of nondairy beverages believed that plant-based drinks helped to reduce the negative impacts of animal production on the environment [4]. Also, there was concern about the health benefits of milk given its saturated fat content [5]. Studies have shown differing effects of milk fat consumption on human health, even though no strong evidence of deleterious impact of moderate milk consumption has been shown [6]. Nevertheless, cow milk is an excellent source of essential nutrients. Based on amino acid profile, the protein quality of cow milk is superior to that of plant-based beverages for human consumption [7]. Another example is the natural content of cow milk vitamin B_12_, also known as cyanocobalamin. Indeed, vitamin B_12_ is solely synthesized by bacteria and archaea when cobalt is not limiting [8]. Bacteria and archaea living in the rumen are able to synthesize vitamin B_12_, which is ultimately secreted in milk [9]. Animal products, especially those from ruminants, are an excellent source of vitamin B_12_, whereas plant-based products, such as soy beverage do not contain it unless they are fortified with the synthetic form of the vitamin. The use of synthetic vitamin B_12_ to fortify soy beverages may not offer the perceived benefit to the consumer. Using a pig model, it was shown that the natural form of vitamin B_12_ in cow milk and cheddar cheese is more bioavailable than the synthetic form [10,11].

The major role of vitamin B_12_ in human health is to prevent megaloblastic anemia and neuropathy [12]. According to the United States Department of Agriculture [13], a 250-mL glass of milk should provide 46% of the recommended dietary allowance (RDA) for humans above 13 years old. Hence, milk is a staple food of major importance in human diet to fulfill the daily vitamin B_12_ requirement. McCarthy et al. [4] concluded that the dairy industry should make efforts to educate consumers on the excellent nutritional value of cow milk. The fact that cow milk naturally contains vitamin B_12_ in contrast with plant-based beverages is a good example of information of which consumers should be made aware.

During the last few years, our research group has focused on refining knowledge regarding vitamin B_12_ concentration in milk, in particular on factors explaining its large concentration variability therein. It has been shown that milk vitamin B_12_ concentrations vary greatly among herds and even among cows housed within the same herd [14,15,16]. Duplessis et al. [14] reported that a 250-mL glass of milk from 100 herds provided between 28 and 61% of the RDA for humans above 13 years old, and that 50% of herds did not reach the 46% threshold. Previous research has reported that this huge variability could be in part due to animal characteristics such as parity and breed [14,17], season [15], milk productivity [14], diet composition [14,15], animal genetics [15,16], and rumen microbiota [18]. These studies aimed to better define factors that cause significant vitamin B_12_ concentration variability in milk in order to be able to offer a product with constant and high vitamin B_12_ concentration, and hence, to increase consumer perception of the benefits of fluid milk consumption.

Although conflicting results exist in the literature, some authors reported that apparent ruminal synthesis (ARS) of vitamin B_12_ is correlated with some indicators of ruminal fermentation [19,20,21]. As the vitamin B_12_ secreted in milk is synthesized by the rumen bacteria, it could be hypothesized that indicators of ruminal fermentation could also be used to estimate milk vitamin B_12_ concentration. Bedding type has been shown to have an impact on milk microbiota [22], but, at present, there is no data demonstrating the impact of environmental factors affecting milk microbiota on vitamin B_12_ concentration in milk. As vitamin B_12_ is only synthesized by bacteria, we hypothesized that any environmental factor modifying milk microbiota could have an impact on vitamin B_12_ concentration in milk. In our attempt to refine knowledge regarding the factors influencing vitamin B_12_ concentrations in milk, the first objective of this study was to assess the impact of indicators of ruminal fermentation (Study 1). A second study was undertaken to assess the impact of recycled manure solid (RMS) bedding and straw bedding on the vitamin B_12_ concentrations of bulk tank milk (Study 2).

## 2. Materials and Methods

The first experiment protocol was approved by the Institutional Committee for Animal Care of the Sherbrooke Research and Development Centre (Agriculture & Agri-Food Canada, Sherbrooke, QC, Canada) and all procedures were conducted according to the code of practice of the National Farm Animal Care Council [23] and the guidelines of the Canadian Council on Animal Care [24]. The second trial was approved by the Animal Care and Use Committee for the Veterinary College of the Université de Montréal (Faculté de médecine vétérinaire, Saint-Hyacinthe, QC, Canada) following the same guidelines of the study 1. In each experiment, significance of results was considered when *p* ≤ 0.05 and a tendency when *p* was between 0.05 and 0.10.

### 2.1. Study 1

#### 2.1.1. Animals and Management

The cows and management of this observational study have already been described by Franco-Lopez et al. [18]. Briefly, all lactating Holstein cows from parity 1 to 6, between 2.9 and 8.0 years of age, and 10 and 392 days in milk (DIM) from the dairy herd at the Sherbrooke Research and Development Centre equipped with rumen cannula (*n* = 45) were sampled from May to August 2018. Herd average milk yield was 10194 kg with 4.22% fat and 3.15% true protein. The Holstein breed was used as it makes up 93% of Canadian cows [25]. This study was first undertaken to evaluate the link between rumen microbiota and milk vitamin B_12_ concentration; the results have been presented elsewhere [18]. A total of 27 cows were sampled twice at 62 ± 5 d apart, while the remaining (*n* = 18) cows were sampled once because they were dry at one of the samplings. This yielded a total of 72 sets of samples. Cows were milked twice daily at 12-h intervals in a milking parlor and housed in a tie-stall barn under 17 h of light per day. Cows were fed once daily as total mixed ration (TMR) at 09:00 h according to one of two lactation rations (Table 1). Cows in group 1 averaged 125 (SD: 89) DIM, ranging from 10 to 346 DIM, while cows in group 2 averaged 307 (SD: 61) DIM, ranging from 230 to 392 DIM.

#### 2.1.2. Sample Collection

**Figure 1:**
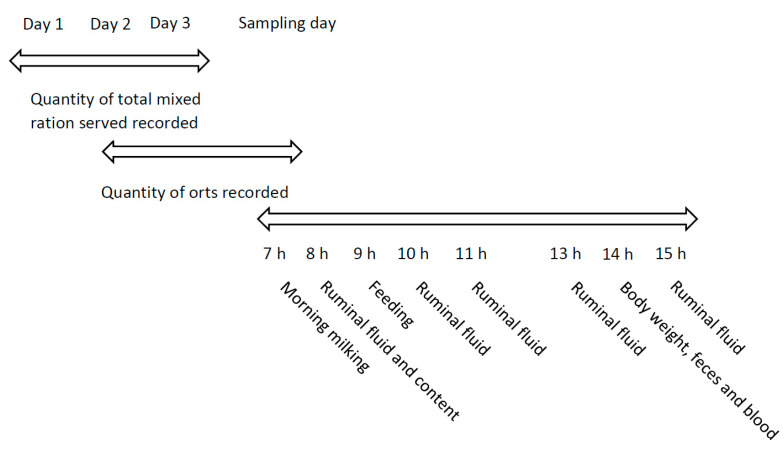
Sampling scheme of study 1. Timeline is based on a 24-h scheme.

The quantity of TMR served and orts were recorded on three consecutive days before the sampling day, and daily dry matter intake (DMI) was calculated (Figure 1). Total mixed ration samples were collected on the same three consecutive days. The body weight (BW) of cows was estimated using heart girth circumference and the equation of Yan et al. [26]. Milk samples were collected from the morning milking at 07:00 h using calibrated inline milk meters. One aliquot was preserved with bronopol and stored at 4 °C. Another aliquot was taken, without bronopol, for vitamin B_12_ analysis. Milk yield was recorded. Whole-rumen digesta samples were collected before the morning meal at five different rumen sites (cranial dorsal, cranial ventral, central, caudal dorsal, and caudal ventral) [27] and manually mixed to get a composite sample. Ruminal fluid was obtained with a 50-mL syringe screwed to a stainless tube with a fine-metal mesh probe (Bar Diamond Inc., Parma, ID, USA) at one end at 0, 1, 2, 4, and 6 h after morning meal distribution. Fifty milliliters of each of the following ruminal locations was collected and mixed to get one composite sample: anterior dorsal, anterior ventral, medium ventral, posterior ventral, and posterior ventral. Ruminal liquid pH was immediately taken after collection (Accumet pH meter; Fisher Scientific, Ottawa, ON, Canada) and aliquots for ammonia N (N-NH_3_) concentration were acidified to pH 2 with 50% H_2_SO_4_. Rectum grab samples of feces were taken 5 h after the morning meal. Blood samples were taken 5 h after the morning meal from the coccygeal vein by venipuncture using a Vacutainer system (Becton, Dickinson and Co., Franklin Lakes, NJ, USA). Tubes with EDTA were put on ice, centrifuged within 30 min for 15 min at 2400× *g* and 4 °C, and plasma was harvested. All samples, unless otherwise specified, were stored at −20 °C until processing.

#### 2.1.3. Laboratory Analyses

Total mixed ration samples were dried in an air-forced oven at 55 °C for 48 h and ground to a particle size of 1 mm (Wiley mill, A. H. Thomas Co., Philadelphia, PA, USA). A composite TMR sample from the three sampling days was then made and analyzed by wet chemistry (SGS Canada, Guelph, ON, Canada) for crude protein (method 990.03; AOAC International [28]), ADF (Ankom Technology, Macedon, NY, USA, Method 12; solutions as in method 973.18; AOAC International [28]), aNDF (Ankom Technology Method 13; solutions as in Van Soest et al. [29] with the inclusion of heat-stable α-amylase), crude fat (Ankom Technology Method 2; AOCS [30]), starch (method 996.11; AOAC International [28]), Ca, P, Mg, K, and Co (inductively coupled plasma; methods 985.01 and 965.09; AOAC International [28]). Net energy of lactation and nonfiber carbohydrates were calculated according to the National Research Council [31].

One aliquot of TMR, whole-rumen digesta and feces samples was put in an air-forced oven at 100 °C for 72 h for dry matter (DM) content determination. Another aliquot of whole-rumen digesta and feces samples was lyophilized (Virtis, SP Scientific, Warminster, PA, USA) and ground to pass through a 1-mm sieve (Wiley mill, A. H. Thomas Co.). Vitamin B_12_ concentration in the latter samples was determined in duplicate as per the method described by Beaudet et al. [19] using a commercial kit (SimulTRAC-S Vitamin B_12_ [Co^57^]/Folate [I^125^], catalog number 06B−254932, MP Biomedicals, Solon, OH, USA). The interassay coefficients of variation for whole-rumen digesta and feces were 3.8 and 3.3%, respectively. Milk samples with bronopol were immediately sent to the Lactanet laboratory (Canadian Network for Dairy Excellence, Sainte-Anne-de-Bellevue, QC, Canada) for fat, protein, and lactose concentration analyses by midinfrared reflectance spectrometry (MilkoScan FT 6000, Foss, Hillerød, Denmark). Vitamin B_12_ concentration in milk and plasma was analyzed in duplicate according to the method described by Duplessis et al. [32], i.e., by radioassay using a commercial kit (SimulTRAC-S Vitamin B_12_ [Co^57^]/Folate [I^125^], MP Biomedicals). The interassay coefficients of variation for milk and plasma were 3.7 and 4.0%, respectively.

The ruminal fluid volatile fatty acid (VFA) profile and ammonia N concentration were analyzed as previously described [33]. Ammonia N concentration was determined using a Varioskan spectrophotometer (type 3001, cat. no 5250010, Thermo Electron Corporation, Vantaa, Finland) at 625 nm, and the VFA profile was obtained with a gas chromatograph (Agilent 6890N; Agilent Technologies Canada Inc., Mississauga, ON, Canada) and a flame detector.

#### 2.1.4. Statistical Analyses

Proc UNIVARIATE of SAS [34] was used to obtain descriptive statistics about the vitamin B_12_ concentrations in different sample types. A first Proc MIXED of SAS was carried out to evaluate the effect of feeding groups as a fixed effect on whole-rumen digesta, feces, plasma, and milk vitamin B_12_ concentrations, and on variables such as DMI, DIM, BW, milk production and components, ruminal ammonia N and VFA concentrations in order to describe the cow population of this study. Cow was added as a random effect to overcome the fact that some cows were sampled twice.

To achieve the objective of the first study, we first had to determine the independent variables to be used in the model. Because liquid ruminal samples were taken several times relative to the meal, there were several possibilities per variable. For instance, propionate concentration at time 0 relative to the meal, minimal and maximal propionate concentrations and average propionate concentration throughout the sampling times could be used as independent variables. For each VFA, these different variables were highly correlated according to the Proc CORR of SAS (Spearman correlation coefficients, ρ > 0.6) and hence they could not be used in the same model. As the vitamin B_12_ concentration in morning milking samples is representative of the last 12 h, between two milkings, we hypothesized that the average of each individual VFA concentration throughout the sampling collection was the most relevant to our model. With the addition of some variables related to the cow, the continuous independent variables considered in our model were as follows: estimated BW, milk yield, milk fat and protein concentrations, DMI, DIM, whole-rumen digesta, feces, and plasma vitamin B_12_ concentrations, and average concentrations of ruminal pH, acetate: propionate ratio, acetate, propionate, butyrate, valerate, isobutyrate, isovalerate, and ammonia N throughout sampling times. To evaluate possible multicollinearity among variables, Proc CORR and Proc REG of SAS were used to obtain Spearman correlation coefficients and variance inflation factor, respectively. A correlation coefficient > 0.6 and a variance inflation factor > 10 suggested multicollinearity [35]. Average concentration of propionate was highly correlated with average concentrations of valerate and isovalerate (ρ > 0.7), and the variance inflation factor of average concentration of propionate was 25. To address this multicollinearity, the methodology described by Duplessis et al. [14] was used, i.e., principal component (PC) analysis was used to reduce the dimensionality of interdependent variables [36]. Proc PRINCOMP of SAS was first used to determine the number of PC to retain for further analysis. Principal components with an Eigenvalue less than 1 were not included for further assessment. Using the number of PC previously determined, Proc FACTOR of SAS was performed with the option VARIMAX rotation to facilitate the PC interpretation. Indeed, with this option, a variable could be associated with a PC when its absolute rotated factor pattern was the largest among the studied PC. The rotated factor pattern had to be higher than 0.5 for a variable to be included in a PC. Each PC was then considered as a continuous independent variable in the following PC regression analysis model:

Y*_i_* = b_0_ + b_1 × 1*i*_ + b_2_PC1*_i_* + b*_3_*PC2*_i_* + … + b_n_PCn*_i_* + v*_i_* + Ɛ*_i_* (1)

where Y*_i_* is the vitamin B_12_ concentration in milk of the *i*-^th^ cow, b_0_ is the fixed intercept; b_1_ is the regression coefficients for feeding group and x_1*i*_ the category for feeding group (i.e., Group 1 and 2) of the *i*-^th^ cow; b_2_ is the regression coefficient of PC1 and PC1 is the score of the first PC of the *i*-^th^ cow and so forth; v*_i_* is the random intercept of the *i*-^th^ cow; and Ɛ*_i_* is the Gaussian error term. The interaction between feeding group and each PC was also examined. Only significant interactions were kept in the final model. Cook’s distance was computed with the INFLUENCE option of SAS, and four records were deleted according to the method of Kaps and Lamberson [35], leaving 68 data for the analysis. The linear relationship between dependent and independent variables was visually assessed with Proc GPLOT of SAS. Residuals were assessed for normality and homoscedasticity. Normality assumption was violated and milk vitamin B_12_ concentration data was then log transformed. Parity was not included in the model because there were only three cows in first parity, and they were at the end of their lactation. Proc REG of SAS was used to determine the pseudo-R^2^ between predicted and observed values of milk vitamin B_12_ concentration obtained from the MIXED model using OUTP option.

### 2.2. Study 2

#### 2.2.1. Herds

The herds involved in this cross-sectional study and cow enrollment have already been described in detail [37,38]. Briefly, herds in Quebec and Eastern Ontario, Canada using RMS bedding and straw bedding for at least six months before the beginning of the study were recruited and visited once from January to July 2018. The main breed of most herds was Holstein. Herds were located within 250 km of the Faculty of Veterinary Medicine of Université de Montréal (Saint-Hyacinthe, QC, Canada). A total of 26 RMS (herd size from 55 to 900 cows, median: 111; freestall, *n* = 20 and tiestall, *n* = 6) and 57 straw herds (herd size from 43 to 229 cows, median: 65; freestall, *n* = 2 and tiestall, *n* = 55) participated in this study. The majority of RMS herds (96%) used RMS bedding which had been composted 3 h to 3 d before its use. Management for straw herds consisted of replacing the bedding every 12 to 24 h. For RMS herds, management used was either shallow or deep bedding. For shallow RMS herds (*n* = 17), bedding was replaced every 12 h, whereas for deep-bedding RMS herds (*n* = 9), it was added from once every other day to once every 8 d.

#### 2.2.2. Sample Collection and Laboratory Analysis

Bulk tank milk samples were taken after 5 min agitation. Samples were then collected via the top of the tank using a sterile straw and syringe, and transferred into a sterile conical tube. If the level of milk in the tank was too low to perform the milk collection as described above, milk was collected in a sterile bag from the outlet valve after discarding a small amount. Milk was then transferred into the conical tube with a sterile syringe. Samples were stored at −20 °C until analysis. The vitamin B_12_ concentrations in those samples were determined as previously explained for study 1. The interassay coefficient of variation was 4.8%.

#### 2.2.3. Statistical Analysis

Proc UNIVARIATE of SAS was used to compute descriptive statistics regarding bulk tank milk vitamin B_12_ concentration. Proc MIXED of SAS was used to evaluate the effect of bedding type as fixed effect on bulk tank milk vitamin B_12_ concentration.

## 3. Results

### 3.1. Study 1

#### 3.1.1. Descriptive Statistics

Based on the 68 samples included for further analysis, vitamin B_12_ concentration in milk averaged 3643 pg/mL (SD: 1497; min to max: 1245 to 8437). Whole-rumen digesta, feces, and plasma vitamin B_12_ concentrations averaged 652 ng/g of DM (SD: 170; min to max: 300 to 1021), 612 ng/g of DM (SD: 125; min to max: 361 to 920), and 215 pg/mL (SD: 70; min to max: 116 to 415), respectively. The dry matter content of whole-rumen digesta and feces averaged 15.7% (SD: 1.5; min to max: 12.0 to 19.2) and 14.1% (SD: 1.6; min to max: 10.4 to 17.2), respectively.

Cows fed with the group 1 ration were associated with lower estimated BW and DIM than those fed the group 2 ration (*p* ≤ 0.0003; Table 2). Cows in group 1 were mainly in early lactation, with 25% having less than 31 DIM and 75% less than 194 DIM. The morning milk yield of cows fed group 1 was almost twice as much as that of group 2 (*p* < 0.0001), but all milk component concentrations were lower for the former (*p* ≤ 0.02). The whole-rumen digesta of cows fed group 1 had greater vitamin B_12_ concentration than that of cows receiving group 2; the opposite was observed for vitamin concentration in milk (*p* ≤ 0.02; Table 2). Regarding ruminal fermentation characteristics, average pH throughout the sampling times was greater by 0.29 for group 2-fed cows compared with those in the other group (*p* < 0.0001). Ruminal liquid concentration of butyrate was greater for cows receiving the ration of group 1 than group 2. There was a tendency for greater proportion of acetate and acetate: propionate ratio for group 2 cows compared with group 1 cows (*p* ≤ 0.08). 

#### 3.1.2. Principal Component Analysis

Six PC having an Eigenvalue > 1 and explaining 75% of the total variation were retained for further analysis (Table 3). Indeed, the PC 6 had an Eigenvalue of 1.04 and the PC 7, 0.82. The first, second and third PC had a variance of 4.8, 2.8, and 2.3, explaining 27, 16, and 13% of the total variation, respectively. Two PC were solely related to ruminal fermentation characteristics (PC1 and PC3). The first PC was positively correlated with propionate and valerate and negatively with the acetate:propionate ratio, whereas the third PC was positively associated with acetate, butyrate, isobutyrate and isovalerate. The second PC pertained to milk production, correlating positively with DIM and morning milk protein concentration and negatively with morning milk yield. The fourth PC was positively associated with estimated cow BW and ruminal pH, and negatively with whole-ruminal digesta vitamin B_12_ concentration. The fifth PC correlated positively with plasma vitamin B_12_ concentration and morning milk fat concentration, and the sixth PC correlated positively with feces vitamin B_12_ concentration, DMI, and ruminal ammonia N concentration. These six PC, each representing a set of interrelated variables, could be considered as independent variables in the multivariable analysis. The rotated pattern coefficients depicted in Table 3 represent the relative contribution of each variable to a PC.

#### 3.1.3. Relationship among Milk Vitamin B_12_ Concentration and Independent Variables

The interaction feeding group × PC1 as well as three other PC had a significant relationship with milk vitamin B_12_ concentration (*p* ≤ 0.05; Table 4). Cows fed group 2 had greater milk vitamin B_12_ concentration when ruminal concentrations of propionate and valerate increased and the acetate:propionate ratio decreased (PC1), whereas the opposite was observed for cows receiving group 1 (Feeding group × PC1 interaction, *p* = 0.002). The PC2 and PC5, related to milk productivity, were positively correlated with milk vitamin B_12_ concentration (*p* ≤ 0.02), meaning that this vitamin in milk increased along with morning milk fat and protein concentrations as DIM increased, even though there was an inverse relationship between milk vitamin B_12_ concentration and morning milk yield. Moreover, milk vitamin B_12_ concentration was positively associated with its plasma concentration (PC5). Finally, vitamin B_12_ concentration in milk was positively correlated with estimated cow BW and ruminal pH and negatively with whole-rumen digesta vitamin B_12_ concentration (PC4; *p* = 0.05). Pseudo-R^2^ was 93%, meaning that the current model explained 93% of the milk vitamin B_12_ variability. When considering only PC2 and PC5 in the model, i.e., PC related to milk productivity and plasma vitamin B_12_ concentration, the pseudo-R^2^ was 88%.

### 3.2. Study 2

**Figure 2:**
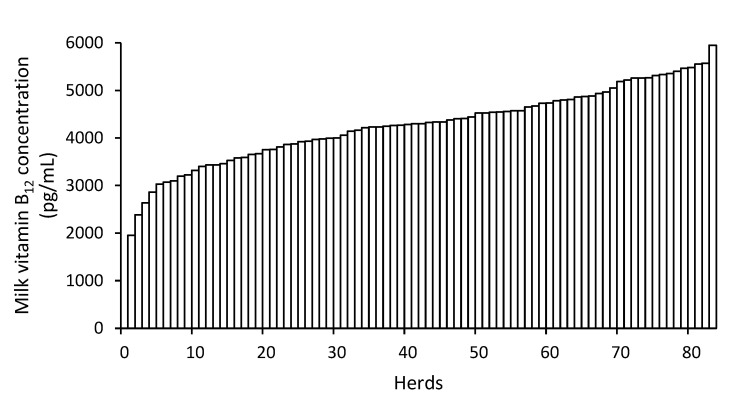
Bulk tank milk vitamin B_12_ concentration among dairy herds.

Bulk tank milk vitamin B_12_ concentration did not differ between RMS and straw bedding herds (*p* = 0.99), and averaged 4276 (SD: 789) pg/mL. The lowest and highest concentrations were 1953 and 5946 pg/mL, respectively (Figure 2).

## 4. Discussion

Milk is considered a staple and complete food. It has been reported that milk and dairy products are the most important sources of daily vitamin B_12_ requirement fulfilment in humans in several countries [39,40,41], and especially in those below the age of 18. According to the National Academy of Sciences [42], vitamin B_12_ RDA for humans above 13 years old is 2.4 µg/d, increasing to 2.6 and 2.8 µg/d for pregnant and lactating women, respectively. A 250-mL milk glass from cows of study 1 could provide between 13 and 88% of the RDA for nongestating and nonlactating humans above 13 years old. Regarding study 2, from 20 to 62% of the RDA could be fulfilled by a 250-mL glass of milk from the involved herds, which is similar to figures presented by Duplessis et al. [14], who reported a RDA fulfillment of between 28 and 61% from a 250-mL glass of milk from 100 commercial dairy herds. The average vitamin B_12_ concentrations in milk reported in study 1 seem low compared to what has been previously reported in the literature [14,16], whereas the average bulk tank milk vitamin B_12_ concentration of study 2 was similar [14]. The results from study 1 could be due to the time of year when the samples were collected. Indeed, it has been reported that milk vitamin B_12_ concentration was lower during the summer months [15,43], which is when experiment 1 was performed. Nevertheless, as commonly reported in the literature, milk vitamin B_12_ concentration was highly variable within cows in the same herd and among herds [14,15,16,17]. Plasma vitamin B_12_ concentrations were similar to those in previous trials [17,44]. To our knowledge, this is the first time that dairy cow whole-rumen digesta and feces vitamin B_12_ concentrations have been reported in the literature.

Cows receiving the group 1 ration in study 1 were typically in early lactation, producing more milk and requiring a diet containing more energy and less fiber. This is supported by lower ruminal pH and acetate:propionate ratio for those cows compared with cows receiving the ration of group 2. Moreover, it has been previously reported on those cows that ruminal microbiota differed between cows fed groups 1 and 2 [18]. Franco-Lopez et al. [18] also found that ruminal microbiota was different between cows with high and low vitamin B_12_ concentrations in whole-rumen digesta. That could explain the significant difference in whole-rumen digesta vitamin B_12_ concentrations between feeding groups. The milk vitamin B_12_ concentration was about 18% greater for cows receiving the ration of group 2 compared with group 1.

The objectives of this paper were to gather more information about factors driving variations in milk vitamin B_12_ concentration. Few studies exist pertaining to the causes of this variation in milk. As milk is an excellent source of vitamin B_12_ for humans who rely on exogenous sources to get this required nutrient, acquiring more knowledge in this regard would help in the development of methods to optimize the secretion of this vitamin in milk. The premise behind study 1 was as follows: as vitamin B_12_ is synthesized by ruminal microbiota, a part of the synthesized vitamin would be used by the cow cells and another part would be secreted in milk; therefore, any factors modifying the vitamin synthesis in the rumen would also modify its secretion in milk. Using diet printouts of 13 commercial herds, we previously observed a positive relationship between milk vitamin B_12_ concentration and dietary percentage of ADF [15]. Then, a study to assess the impact of the actual ration given to dairy cows on milk vitamin B_12_ concentration was conducted on 100 commercial Holstein herds [14]. Using the actual nutrient composition of the diet, we showed that vitamin B_12_ in milk was positively correlated with dietary fiber concentration and negatively correlated with dietary starch and energy concentrations. Moreover, previous results suggested that vitamin B_12_ ARS was correlated with dietary NDF [19,21,45]. This is in line with our results as, in the PC regression analysis, we noted a positive relationship between this vitamin concentration in milk and ruminal pH. It is indeed well recognized that ruminal pH generally increases as dietary forage NDF or physically effective NDF also increases [46,47]. Nevertheless, there is no consensus in the literature about the correlation between vitamin B_12_ ARS and ruminal pH; some authors reported none [19,48], while others observed a negative [21,45] or positive [20] association. This could be explained by the variety of diets offered to dairy cows in those studies, combined with various pH ranges.

In the PC regression analysis, there was a significant feeding group x PC1 interaction effect on milk vitamin B_12_ concentration. PC1 included variables related to ruminal fermentation such as concentrations of propionate and valerate, and the propionate:acetate ratio. With the difference in ration nutrient composition between feeding groups and its subsequent impact on ruminal fermentation, it was not surprising to observe an interaction with feeding group and PC1. However, feeding group might also have a confounding effect with DIM. Duplessis et al. [14] observed a positive relationship between milk vitamin B_12_ concentration and percentage of chopped mixed silage, and a negative relationship with percentage of corn silage in the ration. These results are in accordance with our findings, as cows fed group 2 had greater milk vitamin B_12_ concentration coupled with greater dietary percentage of chopped mixed silage and lower percentage of corn silage than cows fed group 1. These changes in the ration could explain the ruminal fermentation characteristic differences between feeding groups. Franco-Lopez et al. [18] observed, for cows in study 1, that a high concentration of whole-rumen digesta vitamin B_12_ was associated with an increased abundance of the genus *Prevotella* in the rumen. This microorganism is recognized to have a role in propionate synthesis [49]. In the same line, in the current study, cows fed group 1 had greater whole-rumen digesta vitamin B_12_ concentration and a lower acetate:propionate ratio. It is surprising that vitamin B_12_ concentration in milk was negatively correlated with whole-rumen digesta vitamin B_12_ concentration according to PC4. It is noteworthy that whole-rumen digesta vitamin B_12_ concentration is far from being comparable to vitamin B_12_ ARS. Indeed, vitamin ARS is calculated as the difference of vitamin duodenal flow minus vitamin intake [21]. Whole-rumen digesta concentration could be influenced by rumen fill, passage rate and dietary fiber concentration. This could explain the opposite relationship between whole-rumen digesta and milk vitamin B_12_ concentrations.

Vitamin B_12_ concentration in milk was positively associated with estimated cow BW, which is in line with the results of Duplessis et al. [14]. In the current experiment, it could not be determined if the effect of estimated BW was linked to parity. Duplessis et al. [14] observed that milk vitamin B_12_ concentration increased with parity and BW. They concluded that this could be due to the fact that first parity cows, who usually have a lower BW than older cows, use the vitamin for their growth, and hence, less of the vitamin ends up in the milk [14]. Comparable results to those of the present study regarding milk yield and milk fat and protein concentrations were obtained by Duplessis et al. [14], who included over 4300 cows in their model. These authors also reported that milk vitamin B_12_ concentration followed the same typical curve as milk fat and protein concentrations, meaning a concentration nadir of around 50–55 DIM followed by a gradual increase. In the current trial, we did not observe a milk vitamin B_12_ concentration nadir in early lactation, and expDIM, as per Wilmink [50], was not significant in the model and was not included. This could be due to the small number of cows in the current experiment. Nevertheless, the current data suggest that this vitamin concentration in milk increased as lactation progressed, along with milk fat and protein concentrations. Vitamin B_12_ concentration in milk was positively correlated with plasma vitamin B_12_ concentration. It has been reported elsewhere, however, that the Spearman rank correlation coefficient between milk and plasma vitamin B_12_ concentrations of Holstein cows is weak, although significant [17].

The model explained 93% of the variability of milk vitamin B_12_ concentration of study 1, which is excellent. A pseudo-R^2^ of 52% was obtained in a previous study by Duplessis et al. [14] assessing the impact of diet management, milk productivity and cow characteristics on milk vitamin B_12_ concentration variation. However, it is noteworthy that the major difference between study 1 and that of Duplessis et al. [14] was the number of herds involved, i.e., 1 vs. 100 herds, respectively. Involving more herds in a study increases variability due to, among other factors, different management approaches. This is likely the explanation of the higher pseudo-R^2^ in the current experiment. Interestingly, both the current study and that of Duplessis et al. [14] concluded that milk productivity was the main driver of milk vitamin B_12_ concentration variation. Limitations of study 1 include the fact that some cows had to be sampled twice, as there were not sufficient rumen-cannulated cows in the herd. Moreover, the need of rumen-cannulated cows limited the sampling collection to one herd and a relatively small number of animals. Hence, the current results might not be representative of the population. This should be kept in mind when interpreting the results of study 1. Dietary Co concentration was not limiting in the current study as it was above the recommendation of 0.11 mg/kg of DM [31].

Most previous studies evaluating milk vitamin B_12_ concentration variation were directed at factors affecting vitamin synthesis in the rumen, for instance, diet nutrient composition that would first modify vitamin B_12_ synthesis by ruminal bacteria and then its secretion into milk. Recently, evidence has emerged that the udder has a microbiota, although this is still controversial [51]. Nonetheless, it is clear that the teat canal and apex do have a microbiota [51]. Environmental factors such as milking equipment, flies and bedding material could be potential factors affecting mammary microbiota [52]. To the best of our knowledge, there is no information in the literature about potential syntheses or the use of vitamin B_12_ by the mammary microbiota. Using the participating herds of study 2, Gagnon et al. [38] found a higher abundance of Streptococcus spp. and Enterococcus faecalis in bulk milk samples of herds using RMS bedding compared to straw bedding, suggesting a slightly different milk microbiota between these two bedding systems. These species are known to produce vitamin B_12_ [8,53]. However, we did not observe an influence of bedding type on vitamin B_12_ concentration of milk bulk tank samples.

## 5. Conclusions

This paper showed that ruminal fermentation characteristics such as ruminal pH could have an impact on milk vitamin B_12_ concentration. However, the impact of ruminal concentrations of propionate, valerate and the acetate:propionate ratio on milk vitamin B_12_ concentration was different according to the rations given to cows. This suggests that, as expected, the rations modified the ruminal fermentation characteristics and cannot be excluded as a factor influencing milk vitamin B_12_ concentration variability when ruminal fermentation characteristics are also considered. Milk fat and protein concentrations, DIM, estimated cow BW and plasma vitamin B_12_ concentration were all positively associated, and milk yield and whole-rumen digesta vitamin B_12_ concentration were negatively related with milk vitamin B_12_ concentration. The most important factors explaining this were related to milk productivity and plasma vitamin B_12_ concentration; the impact of other studied factors was relatively small. Bedding type, such as RMS and straw beddings, did not have any impact on vitamin B_12_ concentration in bulk tank milk. This study obtained further knowledge regarding factors explaining the huge variation of vitamin B12 concentrations in milk.

## Figures and Tables

**Table 1:** Ingredients and nutrient composition of lactation total mixed ration diets offered to dairy cows ^1^.

Item	Group 1	Group 2
Ingredients (% of dry matter)		
Grass hay	2.6	2.0
Legume-grass silage	23.0	39.2
Corn silage	34.1	26.1
Cracked corn	16.4	15.1
Soybean meal	8.4	9.2
Beet pulp	3.5	
Mineral and vitamin premix ^2^	1.5	1.4
Calcium carbonate	1.0	1.0
Distiller grain (corn)	2.6	1.8
Corn gluten meal	2.6	1.8
Canola meal	1.7	1.2
Micronized soybean	1.7	1.2
Rumen bypass fat	0.97	-
Nutrient composition (% of dry matter unless otherwise specified)		
DM	43.3	42.2
CP	15.0	15.2
ADF	16.1	19.0
aNDF	29.7	35.3
NFC	38.8	35.4
Fat	3.03	2.43
Starch	21.4	17.8
Ca	0.92	0.95
P	0.36	0.39
Mg	0.20	0.22
K	1.31	1.48
Co (mg/kg)	1.88	1.69

Abbreviations: ADF = acid detergent fiber; CP = crude protein; DM = dry matter; aNDF = heat-stable amylase assayed neutral detergent fiber; NFC = nonfiber carbohydrate. ^1^ Adapted from Franco-Lopez et al. [18]. Days in milk averaged 125 SD 89 in group 1 and 307 SD 61 in group 2. ^2^ On a dry matter basis, lactation mineral contained per kg: 93 g of Ca, 49 g of P, 111 g of Na, 82 g of Cl, 11 g of K, 16 g of S, 55 g of Mg, 524 mg of Cu, 1660 mg of Mn, 2968 mg of Zn, 20 mg of Se, 21 mg of Co, 447,811 IU of vitamin A, 56,671 IU of vitamin D, and 2777 IU of vitamin E.

**Table 2:** Cow characteristics, vitamin B_12_ concentrations of whole-rumen digesta, feces, plasma, and milk, and ruminal fermentation characteristics according to the feeding group of the cow ^1^.

Item	Group 1	Group 2	SEM	*p*-Value
Cows (*n*)	53	15		
Cow characteristics				
Dry matter intake (kg)	22.1	21.5	0.9	0.53
Estimated body weight ^2^ (kg)	702	759	13	0.0003
Parity	3.7	3.1	0.3	0.09
Days in milk	125	307	22	<0.0001
Morning milk yield (kg)	18.2	9.3	1.0	<0.0001
Morning milk fat concentration (%)	3.25	3.79	0.20	0.02
Morning milk protein concentration (%)	3.04	3.54	0.06	<0.0001
Morning milk lactose concentration (%)	4.64	4.41	0.06	0.001
Vitamin B_12_ concentration				
Whole-rumen digesta (ng/g)	683	541	41	0.004
Feces (ng/g)	621	582	32	0.29
Plasma (pg/mL)	221	195	18	0.20
Morning milk (pg/mL)	3422	4021	374	0.02
Ruminal fermentation characteristics ^3^				
pH	6.13	6.42	0.04	<0.0001
Volatile fatty acids (mM)				
Acetate	56.2	55.2	2.2	0.70
Propionate	31.2	26.5	3.0	0.18
Butyrate	11.8	10.3	0.6	0.02
Isobutyrate	0.8	0.8	0.1	0.84
Valerate	1.2	1.2	0.1	0.67
Isovalerate	1.7	1.5	0.1	0.18
Total	102.9	95.5	4.9	0.18
Volatile fatty acids (mol/100 mol)				
Acetate	55.3	58.5	1.4	0.06
Propionate	29.3	27.0	1.9	0.31
Butyrate	11.8	10.9	0.7	0.25
Isobutyrate	0.9	0.9	0.1	0.37
Valerate	1.2	1.2	0.1	0.88
Isovalerate	1.6	1.5	0.1	0.36
Acetate: propionate ratio	2.0	2.5	0.2	0.08
Ammonia (mM)	10.7	11.0	0.9	0.80

^1^ Analysis after suppression of influential data using Cook’s distance. ^2^ According to the equation of Yan et al. [26]. ^3^ Averaged records from 0, 1, 2, 4, and 6 h relative to the meal presented.

**Table 3:** Rotated pattern coefficients ^1^ of the principal component (PC) analysis.

Variables	PC1	PC2	PC3	PC4	PC5	PC6
Cow characteristics						
Dry matter intake	−28	−5	−21	27	−47	59
Estimated body weight	−11	5	−15	77	5	6
Days in milk	−19	81	7	26	−11	8
Morning milk yield	−5	−75	−6	−22	−18	30
Morning milk fat concentration	3	46	−6	4	78	4
Morning milk protein concentration	−30	78	−27	2	−5	8
Vitamin B_12_ concentration						
Whole-rumen digesta	−8	−34	24	−54	34	−2
Feces	10	−12	22	−10	21	74
Plasma	3	−16	6	4	78	4
Ruminal fermentation characteristics						
pH	−13	29	−7	70	12	−26
Volatile fatty acids (m*M*)						
Acetate	31	−1	84	−14	12	5
Propionate	91	−7	22	−14	12	−2
Butyrate	−7	−17	69	−35	−33	6
Isobutyrate	−58	3	63	6	21	21
Valerate	85	−17	24	9	−2	−3
Isovalerate	56	0	67	−19	19	20
Acetate: propionate ratio	−93	13	11	17	−5	7
Ammonia (m*M*)	−41	31	17	−20	2	54

^1^ Multiplied by 100 and rounded to the closest integer.

**Table 4:** Results from the principal component (PC) regression analysis regarding the relationship between milk vitamin B_12_ concentration and ruminal fermentation characteristics, as well as milk productivity and cow characteristics ^1^.

Variables	Estimate	SEM	*p*-Value
Intercept	7.99	0.12	<0.0001
Feeding Group			
Group 1	0.15	0.14	0.29
Group 2	0		
Feeding group x PC1			0.002
Group 1	−0.08	0.04	0.05
Group 2	0.18	0.06	0.006
PC1	0		0.14
PC2	0.15	0.06	0.02
PC3	0.04	0.04	0.28
PC4	0.12	0.06	0.05
PC5	0.24	0.04	<0.0001
PC6	−0.07	0.04	0.12

^1^ The analysis was performed using the log-transformed milk vitamin B_12_ concentration as dependent variable. Hence, estimates and SEM are presented according to the log scale.

## Data Availability

Data is contained within the article.
